# Supplementary material for: Modeling glioblastoma heterogeneity as a dynamic network of cell states
Source: Mol Syst Biol. 2021 Sep 16;17(9):e10105. doi: 10.15252/msb.202010105 (PMC8444284; doi:10.15252/msb.202010105)
Supplement: Supplementary file 6 — Source Data for Figure 5 [file MSB-17-e10105-s004.zip › Figure5A_sourcedata/GSEA_3071/hallmarks_stateVI.GseaPreranked.1623742557031/HALLMARK_ANGIOGENESIS.html]

Details for gene set HALLMARK\_ANGIOGENESIS[GSEA]

|  || Dataset | state63071 |
| Phenotype | NoPhenotypeAvailable |
| Upregulated in class | na\_neg |
| GeneSet | HALLMARK\_ANGIOGENESIS |
| Enrichment Score (ES) | -0.5107284 |
| Normalized Enrichment Score (NES) | -1.6579216 |
| Nominal p-value | 0.033189032 |
| FDR q-value | 0.13891375 |
| FWER p-Value | 0.605 |
Table: GSEA Results Summary

  

Fig 1: Enrichment plot: HALLMARK\_ANGIOGENESIS      
 Profile of the Running ES Score & Positions of GeneSet Members on the Rank Ordered List

  

| PROBE | GENE SYMBOL | GENE\_TITLE | RANK IN GENE LIST | RANK METRIC SCORE | RUNNING ES | CORE ENRICHMENT || 1 | JAG1 |  |  | 876 | 0.365 | -0.2010 | No |
| 2 | CCND2 |  |  | 1064 | 0.335 | -0.2151 | No |
| 3 | JAG2 |  |  | 1281 | 0.306 | -0.2406 | No |
| 4 | VEGFA |  |  | 2104 | -0.298 | -0.4341 | No |
| 5 | COL5A2 |  |  | 2160 | -0.307 | -0.4150 | No |
| 6 | PTK2 |  |  | 2347 | -0.343 | -0.4280 | No |
| 7 | LRPAP1 |  |  | 2392 | -0.352 | -0.4008 | No |
| 8 | ITGAV |  |  | 2476 | -0.371 | -0.3822 | No |
| 9 | LUM |  |  | 2775 | -0.448 | -0.4144 | No |
| 10 | TNFRSF21 |  |  | 3125 | -0.600 | -0.4436 | Yes |
| 11 | FGFR1 |  |  | 3204 | -0.672 | -0.3901 | Yes |
| 12 | STC1 |  |  | 3393 | -0.880 | -0.3436 | Yes |
| 13 | COL3A1 |  |  | 3430 | -0.960 | -0.2463 | Yes |
| 14 | SPP1 |  |  | 3541 | -1.286 | -0.1330 | Yes |
| 15 | APP |  |  | 3562 | -1.427 | 0.0210 | Yes |
Table: GSEA details [plain text format]

  

Fig 2: HALLMARK\_ANGIOGENESIS: Random ES distribution      
 Gene set null distribution of ES for **HALLMARK\_ANGIOGENESIS**
